# Non-negative matrix factorization by maximizing correntropy for cancer clustering

**DOI:** 10.1186/1471-2105-14-107

**Published:** 2013-03-24

**Authors:** Jim Jing-Yan Wang, Xiaolei Wang, Xin Gao

**Affiliations:** 1 Computer, Electrical and Mathematical Sciences and Engineering Division, King Abdullah University of Science and Technology (KAUST), Thuwal 23955-6900, Saudi Arabia; 2 Computational Bioscience Research Center, King Abdullah University of Science and Technology (KAUST), Thuwal 23955-6900, Saudi Arabia

## Abstract

**Background:**

Non-negative matrix factorization (NMF) has been shown to be a powerful tool for clustering gene expression data, which are widely used to classify cancers. NMF aims to find two non-negative matrices whose product closely approximates the original matrix. Traditional NMF methods minimize either the *l*_2_ norm or the Kullback-Leibler distance between the product of the two matrices and the original matrix. Correntropy was recently shown to be an effective similarity measurement due to its stability to outliers or noise.

**Results:**

We propose a maximum correntropy criterion (MCC)-based NMF method (NMF-MCC) for gene expression data-based cancer clustering. Instead of minimizing the *l*_2_ norm or the Kullback-Leibler distance, NMF-MCC maximizes the correntropy between the product of the two matrices and the original matrix. The optimization problem can be solved by an expectation conditional maximization algorithm.

**Conclusions:**

Extensive experiments on six cancer benchmark sets demonstrate that the proposed method is significantly more accurate than the state-of-the-art methods in cancer clustering.

## Background

Because cancer has been a leading cause of death in the world for several decades, the classification of cancers is becoming more and more important to cancer treatment and prognosis [1,2]. With advances in DNA microarray technology, it is now possible to monitor the expression levels of a large number of genes at the same time. There have been a variety of studies on analyzing DNA microarray data for cancer class discovery [3-5]. Such methods are demonstrated to outperform the traditional, morphological appearance-based cancer classification methods. In such studies, different cancer classes are discriminated by their corresponding gene expression profiles [1].

Several clustering algorithms have been used to identify groups of similar expressed genes. Non-negative matrix factorization (NMF) was recently introduced to analyze gene expression data and this method demonstrated superior performance in terms of both accuracy and stability [6-8]. Gao and Church [3] reported an effective unsupervised method for cancer clustering with gene expression profiles via sparse NMF (SNMF). Carmona et al. [9] presented a methodology that was able to cluster closely related genes and conditions in sub-portions of the data based on non-smooth non-negative matrix factorization (nsNMF), which was able to identify localized patterns in large datasets. Zheng et al. [5,7] applied penalized matrix decomposition (PMD) to extract meta-samples from gene expression data, which could captured the inherent structures of samples that belonged to the same class.

NMF approximates a given gene data matrix, *X*, as a product of two low-rank nonnegative matrices, *H* and *W*, as *X*≈*HW*. This is usually formulated as an optimization problem, where the objective function is to minimize either the *l*_2_ norm or the Kullback-Leibler (KL) distance [10] between *X* and *HW*. Most of the improved NMF algorithms are also based on the minimization of these two distances while adding the sparseness term [3], the graph regularization term [11], etc. Sandler and Lindenbaum [12] argued that measuring the dissimilarity of *W* and *HW* by either the *l*_2_ norm or the KL distance, even with additional bias terms, was inappropriate in computer vision applications due to the nature of errors in images. Sandler and Lindenbaum [12] proposed a novel NMF with earth mover’s distance (EMD) metric by minimizing the EMD error between *X* and *HW*. The proposed NMF-EMD algorithm demonstrated significantly improved performance in two challenging computer vision tasks, i.e., texture classification and face recognition. Liu et al. [4] tested a family of NMF algorithms using *α*-divergence with different *α* values as dissimilarities between *X* and *HW* for clustering cancer gene expression data.

It is widely acknowledged that DNA microarry data contain many types of noise, especially experimental noise. Recently, correntropy was shown to be an effective similarity measurement in information theory due to its stability to outliers or noise [13]. However, it has not been used in the analysis of microarray data. In this paper, we propose a novel form of NMF that maximizes the correntropy. We introduce a new NMF algorithm with a maximum correntropy criterion (MCC) [13] for the gene expression data-based cancer clustering problem. We call it NMF-MCC. The goal of NMF-MCC is to find a meta-sample matrix, *H*, and a coding matrix, *W*, such that the gene expression data matrix, *X*, is as correlative to the product of *H* and *W* as possible under MCC.

### Related works

He et al. [13] recently developed a face recognition algorithm, correntropy-based sparse representation (CESR), based on MCC. CESR tries to find a group of sparse combination coefficients to maximize the correntropy between the facial image vector and the linear combination of faces in the database. He et al. [13] demonstrated that CESR was much more effective in dealing with the occlusion and corruption problems of face recognition than the state-of-the-art methods. However, CESR learns only the combination coefficients while the basis faces (the faces in the database) are fixed. Comparing to CESR, NMF-MCC can learn both the combination coefficients and the basis vectors jointly, which allows the algorithm to obtain more basis vectors for better representation of the data points. Zafeiriou and Petrou [14] addressed the problem of NMF with kernel functions instead of inner products and proposed the projected gradient kernel nonnegative matrix factorization (PGK-NMF) algorithm. Both NMF-MCC and PGK-NMF employ kernel functions to map the linear data space to a non-linear space. However, as we show later, NMF-MCC computes different kernels for different features, while PGK-NMF computes a single kernel for the whole feature vector. Thus, NMF-MCC allows the algorithm to assign different weights to different features and emphasizes the discriminant features with high weights, thus achieving feature selection. In contrast, like most kernel based methods, PGK-NMF simply replaces the inner product by the kernel-function and treats the features equally, thus there is no feature selection function.

## Methods

In this section, we first briefly introduce the traditional NMF method. We then propose our novel NMF-MCC algorithm by maximizing the correntropy in NMF. We further propose a expectation conditional maximization-based approach to solve the optimization problem.

### Nonnegative matrix factorization

NMF is a matrix factorization algorithm that focuses on the analysis of data matrices whose elements are nonnegative. Consider a gene expression dataset that consists of *D* genes in *N* samples. We denote it by a matrix $X=[x_{1},\cdots \;,x_{N}]\in {ℜ}^{D\times N}$ of size *D*×*N*, and each column of *X* is a sample vector containing *D* genes. NMF aims to find two non-negative matrices, $H=[h_{\text{dk}}]\in {ℜ}^{D\times K}$ and $W=[w_{\text{kn}}]\in {ℜ}^{K\times N}$, whose product closely approximates the original matrix *X*:

(1) X≈HW.

**Figure 1:**
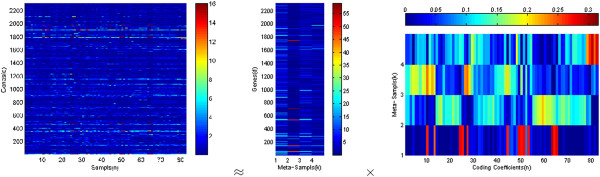
**The *l***_***2 ***_**norm distance-based non-negative matrix factorization on the SRBCT dataset [29].** The gene expression data matrix, *X*, is factorized as the product of the meta-sample matrix, *H*, and the coding matrix, *W*.

Matrix *H* is of size *D*×*K*, with each of the *K* columns defining a meta-sample and each entry, *h*_*dk*_, in *H* representing the expression level of gene *d* over meta-sample *k*. Matrix *W* is of size *K*×*N*, with each of the *n* columns representing the meta-sample expression pattern of the corresponding sample, and each entry, *w*_*kn*_, representing the coefficient of meta-sample *k* over sample *n*. Figure 1 shows an example of the factorization of a gene expression matrix *X* with *D*=2308 genes and *N*=83 samples as the product of the meta-sample matrix *H* with *K*=4 meta-samples and the coding matrix *W*.

The factorization is quantified by an objective function that minimizes some distance measure, such as: 

•***l***_***2***_**norm distance**: One simple measure is the square of the *l*_2_ norm distance (also known as the Frobenius norm or the Euclidean distance) between two matrices, which is defined as:

(2) $$\begin{array}{l} F^{l_{2}}=\overset{D}{\underset{d=1}{\sum }}\overset{N}{\underset{n=1}{\sum }}{\left(X_{\text{dn}}-\overset{K}{\underset{k=1}{\sum }}H_{\text{dk}}W_{\text{kn}}\right)}^{2}. \end{array}$$

•**Kullback - Leibler (KL) divergence**: The second one is the divergence between two matrices [10], which is defined as:

(3) $$F^{\text{KL}}=\overset{D}{\underset{d=1}{\sum }}\overset{N}{\underset{n=1}{\sum }}\left(X_{\text{dn}}\text{ln}\frac{X_{\text{dn}}}{{\left(\text{HW}\right)}_{\text{dn}}}-X_{\text{dn}}+{\left(\text{HW}\right)}_{\text{nd}}\right).$$

### Maximum correntropy criterion for NMF

Another thing that has to be changed is that the definition of correntropy is not subject to the kernel being Gaussian as they seem to imply through the text, so for instance when they define they can say E(k(x-y)) and one of the common choices of k is the Gaussian kernel giving....

Correntropy is a nonlinear similarity measure between two random variables, *x* and *y*[13,15,16], defined as

(4) $$V_{\sigma }(x,y)=E[k_{\sigma }(x-y)],$$

where *k*_*σ*_ is a kernel that satisfies the Mercer theory and *E*[·] is the expectation. One of the common choices of *k*_*σ*_ is the Gaussian kernel given as $k_{\sigma }(x-y)=\text{exp}(-\frac{{\left(x-y\right)}^{2}}{2\sigma^{2}})$.

In practice, the joint probability density function of *x* and *y* is unknown and only a finite amount of data {(*x*_*i*_,*y*_*i*_)},*i*=1,⋯,*I* is available. Therefore, the sample correntropy is estimated by

(5) $${\hat{V}}_{\sigma }(x,y)=\frac{1}{I}\overset{I}{\underset{i=1}{\sum }}k_{\sigma }(x_{i}-y_{i}),$$

Based on Eq. (5), a general similarity measurement between any two discrete gene expression vectors was proposed [17]. They introduced the correntropy induced metric (CIM) for any two gene sample vectors *x*=[*x*_1_,⋯,*x*_*D*_]^⊤^ and *y*=[*y*_1_,⋯,*y*_*D*_]^⊤^, as:

(6) $$\begin{array}{l} \text{CIM}(x,y)={\left(k_{\sigma }(0)+\frac{1}{D}\overset{D}{\underset{d=1}{\sum }}k_{\sigma }(x_{d}-y_{d})\right)}^{\frac{1}{2}}\\ \;={\left(k_{\sigma }(0)+\frac{1}{D}\overset{D}{\underset{d=1}{\sum }}k_{\sigma }(e_{d})\right)}^{\frac{1}{2}}, \end{array}$$

where *e*_*d*_=*x*_*d*_−*y*_*d*_ is defined as the error. For adaptive systems, we can define the maximum correntropy criterion (MCC) [18] as

(7) $$\begin{array}{l} \underset{\Theta }{\max}\overset{D}{\underset{d=1}{\sum }}k_{\sigma }(x_{d}-y_{d}),\\ k_{\sigma }(x_{d}-y_{d})=\text{exp}\;\left[-\frac{{\left(x_{d}-y_{d}\right)}^{2}}{2\sigma^{2}}\right] \end{array}$$

where *Θ* is a parameter to be specified later. We must notice the difference between MCC and common kernel criterion used in [14]. The Gaussian kernel function of vectors *x* and *y* is defined as

(8) $$\begin{array}{l} k_{\sigma }(x-y)=\text{exp}\;\left(-\frac{||x-y|{|}^{2}}{2\sigma^{2}}\right)\\ \;=\text{exp}\;\left[-\frac{\sum_{d=1}^{D}{\left(x_{d}-y_{d}\right)}^{2}}{2\sigma^{2}}\right]. \end{array}$$

We can see that the kernel is applied to the entire feature vector, *x*, and each feature *x*_*d*_,*d*=1⋯,*D* is treated equally with the same kernel parameter. However, in (7), kernel functions are applied to different functions. This can allow the algorithm to learn different kernel parameters as we will introduce later. In this way, we can assign different weights to different features and thus implement feature selection.

Our goal is to find a meta-sample matrix, *H*, and a coding matrix, *W*, such that *HW* is as correlative to *X* as possible under MCC as described in Eq. (7). To extend MCC from vector space *R*^*D*^ to matrix space *R*^*D*×*N*^, we replace *e*_*d*_=(*x*_*d*_−*y*_*d*_) with the *l*_2_ norm distance between the samples of *X* and *Y*=*HW* as $e_{d}=\sqrt{\sum_{n=1}^{N}{\left(x_{\text{dn}}-y_{\text{dn}}\right)}^{2}}$, where *y*_*dn*_ is the (*d*,*n*)-th item of *Y*, and $y_{\text{dn}}=\sum_{k=1}^{K}h_{\text{dk}}w_{\text{kn}}$. Moreover, the factorization system parameter should be set to *Θ*=(*H*,*W*) under the framework of NMF-MCC. By substituting newly defined *e*_*d*_ and *Θ* to (7), we can formulate the problem of NMF-MCC as the following optimization problem:

(9) $$\begin{array}{l} \underset{H,W}{\text{max}}\;F(H,W)\\ \mathrm{s.t.}\;H\geq 0,\;W\geq 0.\\ F(H,W)=\overset{D}{\underset{d=1}{\sum }}k_{\sigma }\left(e_{d}\right)\\ =\overset{D}{\underset{d=1}{\sum }}k_{\sigma }\left(\sqrt{\overset{N}{\underset{n=1}{\sum }}{\left(x_{\text{dn}}-\overset{K}{\underset{k=1}{\sum }}h_{\text{dk}}w_{\text{kn}}\right)}^{2}}\right)\\ =\overset{D}{\underset{d=1}{\sum }}\text{exp}\;\left(-\frac{\sum_{n=1}^{N}{\left(x_{\text{dn}}-\sum_{k=1}^{K}h_{\text{dk}}w_{\text{kn}}\right)}^{2}}{2\sigma^{2}}\right). \end{array}$$

We should notice the significant difference between NMF-MCC and CESR. As a supervised learning algorithm, the CESR represents a test data point, *x*_*t*_, as a linear combination of all the the training data points as $x_{t}\approx \sum_{n=1}^{N}x_{n}w_{\text{nt}}=Xw_{t}$ and *w*_*t*_=[*w*_1*t*_,⋯,*w*_*Nt*_]^⊤^ is the combination coefficient vector. CESR aims to find the optimal *w*_*t*_ to maximize the correntropy between *x*_*t*_ and *Xw*_*t*_. Similarly, NMF-MCC also tries to represent a data point *x*_*n*_ as a linear combination of some basis vectors as $x_{n}\approx \sum_{k=1}^{K}h_{k}w_{\text{kn}}=Xw_{n}$ and *w*_*n*_=[*w*_1*n*_,⋯,*w*_*Kn*_]^⊤^ is the combination coefficient vector. Differently from CESR, NMF-MCC aims to find not only the optimal *w*_*n*_ but also the basis vectors in *H* to maximize the correntropy between *x*_*n*_ and *Hw*_*n*_, *n*=1,⋯,*N*. The internal difference between NMF-MCC and CESR lies in whether to learn basis vectors or not.

In order to solve the optimization problem, we recognize that the expectation conditional maximization (ECM) method [19] can be applied. Based on the theory of convex conjugate functions [20], we can derive the following proposition that forms the basis to solve the optimization problem in (9):

#### Proposition 1

*There exists a convex conjugate function of g*(*z*,*σ*) *such that*

(10) $$\begin{array}{l} g(z,\sigma )=\underset{\varrho \in {ℜ}^{-}}{\text{sup}}\left(\varrho \frac{||z|{|}^{2}}{\sigma^{2}}-\varphi (\varrho )\right) \end{array}$$

*and for a fixed z, the supremum is reached at ϱ=−g*(*z*,*σ*).

By substituting Eq. (10) into (9), we have the augmented objective function in an enlarged parameter space

(11) $$\begin{array}{l} \;\underset{H,W,\rho }{\text{max}}\;\hat{F}(H,W,\rho )\\ \;\mathrm{s.t.}\;H\geq 0,\;W\geq 0.\\ \;\hat{F}(H,W,\rho )\;=\;\overset{D}{\underset{d=1}{\sum }}\left(\rho_{d}\overset{N}{\underset{n=1}{\sum }}{\left(x_{\text{dn}}\;-\;\overset{K}{\underset{k=1}{\sum }}h_{\text{dk}}w_{\text{kn}}\right)}^{2}\;-\;\varphi (\rho_{d})\right), \end{array}$$

where superscript *φ* is the convex conjugate function *φ* of *g*(*z*) defined in Proposition 1, and ***ρ***=[*ρ*_1_,⋯,*ρ*_*D*_]^⊤^ are the auxiliary variables.

According to Proposition 1, for fixed *H* and *W*, the following equation holds:

(12) $$\begin{array}{ll} F(H,W)=\underset{\rho }{\text{max}} & \hat{F}(H,W,\rho ). \end{array}$$

It follows that

(13) $$\begin{array}{l} \underset{H,W}{\text{max}}F(H,W)=\underset{H,W}{\text{max}}\left[\underset{\rho }{\text{max}}\hat{F}(H,W,\rho )\right]\\ \;=\underset{H,W,\rho }{\text{max}}\hat{F}(H,W,\rho ). \end{array}$$

That is, maximizing *F*(*H*,*W*) is equivalent to maximizing the augmented function $\hat{F}(H,W,\rho )$.

### The NMF-MCC Algorithm

**Figure 2:**
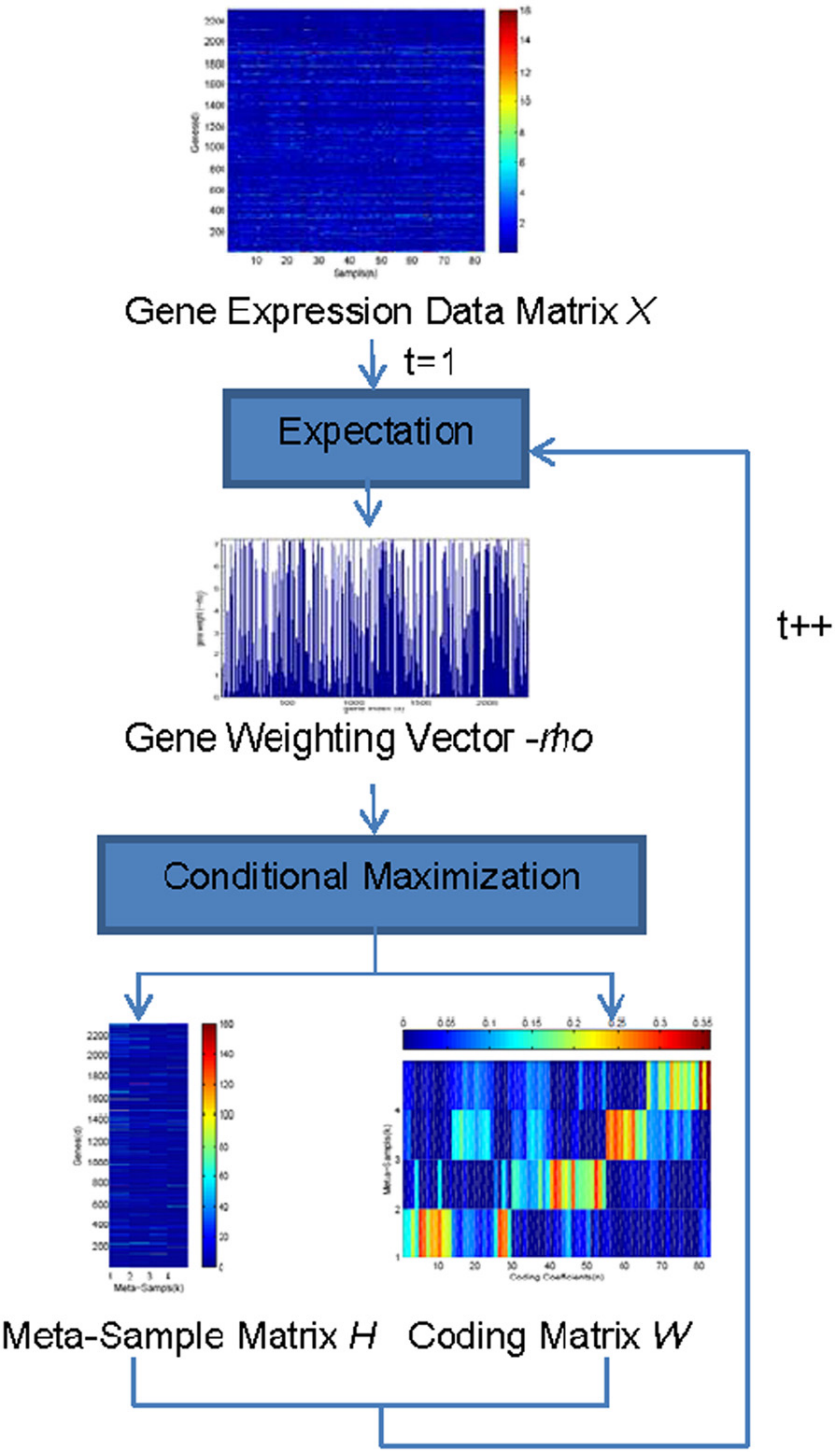
Outline of the ECM-based NMF-MCC algorithm.

The traditional NMF can be solved by the expectation-maximization (EM) algorithm [21]. However, in the case of MCC-based NMF, EM must be replaced by ECM because there is more than one parameter. Figure 2 shows the outline of ECM, which is described in more detail below. 

1. **E-Step**: Compute ***ρ*** given the current estimations of the meta-sample matrix *H* and the coding matrix *W* as:

(14) $$\begin{array}{l} \rho_{d}^{t}=-g\;\left(\sqrt{\overset{N}{\underset{n=1}{\sum }}{\left(x_{\text{dn}}-\overset{K}{\underset{k=1}{\sum }}h_{\text{dk}}^{t}w_{\text{kn}}^{t}\right)}^{2}},\sigma^{t}\right), \end{array}$$

where *t* means the *t*-th iteration. In this study, the kernel size (bandwidth) *σ*^2^^*t*^ is computed by

(15) $$\begin{array}{l} {\sigma^{2}}^{t}=\frac{\theta }{2D}\overset{D}{\underset{d=1}{\sum }}\overset{N}{\underset{n=1}{\sum }}{\left(x_{\text{dn}}-\overset{K}{\underset{k=1}{\sum }}h_{\text{dk}}^{t}w_{\text{kn}}^{t}\right)}^{2}, \end{array}$$

where *Θ* is a parameter to control the sparseness of $\rho_{d}^{t}$.

2. **CM-steps**: In the CM-step, given $\rho_{d}^{t}$, we try to optimize the following function respect to *H* and *W*:

(16) $$\begin{array}{l} \;(H^{t+1},W^{t+1})=\underset{H,W}{\text{argmax}}\overset{D}{\underset{d=1}{\sum }}\left(\rho_{d}^{t}\overset{N}{\underset{n=1}{\sum }}{\left(x_{\text{dn}}-\overset{K}{\underset{k=1}{\sum }}h_{\text{dk}}w_{\text{kn}}\right)}^{2}\right)\\ \;=\underset{H,W}{\text{argmax}}\;\text{Trac}\left[{\left(X\;-\;\text{HW}\right)}^{⊤}\text{diag}\;(\rho^{t})(X\;-\;\text{HW})\right]\\ \;\mathrm{s.t.}\;H\geq 0,\;W\geq 0, \end{array}$$

where *diag*(·) is an operator that converts the vector ***ρ*** to a diagonal matrix.

By introducing a dual objective function,

(17) $$\begin{array}{l} \;O(H,W)=\text{Trac}\;\left[{\left(X-\text{HW}\right)}^{⊤}\text{diag}(-\rho^{t})(X-\text{HW})\right]\\ \;=\text{Trac}\;\left[X^{⊤}\text{diag}(-\rho^{t})X\right]\;\;-\;2\text{Trac}\;\left[X^{⊤}\text{diag}(-\rho^{t})\text{HW}\right]\\ \;+\text{Trac}\;\left[W^{⊤}H^{⊤}\text{diag}(-\rho^{t})\text{HW}\right], \end{array}$$

the optimal problem in (16) can be reformulated as the following dual problem:

(18) $$\begin{array}{l} \left(H^{t+1},W^{t+1})=\underset{H,W}{\text{argmin}}\;O(H,W\right)\\ \;\mathrm{s.t.}\;H\geq 0,\;W\geq 0. \end{array}$$

Let *ϕ*_*dk*_ and *ψ*_*kn*_ be the Lagrange multiplier for constraints *h*_*dk*_≥0 and *w*_*kn*_≥0, respectively, and *Φ*=[*ϕ*_*dk*_] and *Ψ*=[*ψ*_*kn*_]. The Lagrange ℒ is

(19) $$\begin{array}{l} \;ℒ=\text{Trac}\;\left[X^{⊤}\text{diag}(-\rho^{t})X\right]-2\text{Trac}\;\left[X^{⊤}\text{diag}(-\rho^{t})\text{HW}\right]\\ \;+\text{Trac}\;\left[W^{⊤}H^{⊤}\text{diag}(-\rho^{t})\text{HW}\right]+\text{Trac}\;\left[\Phi H^{⊤}\right]\\ \;+\text{Trac}\;\left[\Psi W^{⊤}\right]. \end{array}$$

The partial derivatives of ℒ with respect to *H* and *W* are

(20) $$\begin{array}{ll} \frac{\partial ℒ}{\mathrm{\partial H}}= & -2\text{diag}(-\rho^{t})XW^{⊤}+2\text{diag}(-\rho^{t})\text{HW}W^{⊤}\;+\;\Phi \end{array}$$

and

(21) $$\begin{array}{l} \frac{\partial ℒ}{\mathrm{\partial W}}=-2H^{⊤}\text{diag}(-\rho^{t})X+2H^{⊤}\text{diag}(-\rho^{t})\text{HW}+\Psi \end{array}$$

Using the Karush-Kuhn-Tucker optimal conditions, i.e., *ϕ*_*dk*_*h*_*dk*_=0 and *ψ*_*kn*_*w*_*kn*_=0, we get the following equations for *h*_*dk*_ and *w*_*kn*_:

(22) $$\begin{array}{l} -2{\left(\text{diag}(-\rho^{t})XW^{⊤}\right)}_{\text{dk}}h_{\text{dk}}\\ +2{\left(\text{diag}(-\rho^{t})\text{HW}W^{⊤}\right)}_{\text{dk}}h_{\text{dk}}=0 \end{array}$$

and

(23) $$\begin{array}{l} -2{\left(H^{⊤}\text{diag}(-\rho^{t})X\right)}_{\text{kn}}w_{\text{kn}}\\ +2{\left(H^{⊤}\text{diag}(-\rho^{t})\text{HW}\right)}_{\text{kn}}w_{\text{kn}}=0 \end{array}$$

These equations lead to the following updating rules to maximize the expectation in (13). 

••The meta-sample matrix *H*, conditioned on the coding matrix *W*:

(24) $$\begin{array}{l} h_{\text{dk}}^{t+1}\leftarrow h_{\text{dk}}^{t}\frac{{\left(\text{diag}(-\rho^{t})X{W^{t}}^{⊤}\right)}_{\text{dk}}}{{\left(\text{diag}(-\rho^{t})H^{t}W^{t}{W^{t}}^{⊤}\right)}_{\text{dk}}} \end{array}$$

••The coding matrix *W* conditioned on the newly estimated meta-sample matrix *H*^*t*+1^:

(25) $$\begin{array}{l} w_{\text{kn}}^{t+1}\leftarrow w_{\text{kn}}^{t}\frac{{\left({H^{t+1}}^{⊤}\text{diag}(-\rho^{t})X\right)}_{\text{kn}}}{{\left({H^{t+1}}^{⊤}\text{diag}(-\rho^{t})H^{t+1}W^{t}\right)}_{\text{kn}}} \end{array}$$

We should note that if we exchange the numerator and denominator in (24) and (25), new update formulas will be yield. The new update rules are dual for (24) and (25), and our experimental results show that the dual update rules achieve similar clustering performances as (24) and (25).

Algorithm 1 summarizes the optimization procedure.

### Algorithm 1 NMF-MCC Algorithm

### Proof of convergence

In this section, we will prove that the objective function in (16) is nonincreasing under the updating rules in (24) and (25).

#### Theorem 1

The objective function in (16) is nonincreasing under the update rules (24) and (25).

To prove the above theorem, we first define an auxiliary function.

#### Definition 1

*G*(*w*,*w*^′^) is an auxiliary function for *F*(*w*) if the conditions

(26) $$G(w,w^{\prime })\geq F(w),\;G(w,w)=F(w)$$

are satisfied.

The auxiliary function is quite useful because of the following lemma:

#### Lemma 1

If *G* is an auxiliary function of *F*, then *F* is nonincreasing under the update

(27) $$w^{t+1}=\underset{w}{\text{argmin}}G(w,w^{t}).$$

We refer the readers to [22] for the proof of this lemma. Now, we show that the updating rule of (25) is exactly the update in (27) with a proper auxiliary function. We denote the objective function in (16) as *O*:

(28) $$\begin{array}{l} O=\overset{D}{\underset{d=1}{\sum }}\left(\rho_{d}\overset{N}{\underset{n=1}{\sum }}{\left(x_{\text{dn}}-\overset{K}{\underset{k=1}{\sum }}h_{\text{dk}}w_{\text{kn}}\right)}^{2}\right)\\ \;=\text{Trac}\left[{\left(X-\text{HW}\right)}^{⊤}\text{diag}\;(\rho^{t})(X-\text{HW})\right]. \end{array}$$

Considering any element, *w*_*kn*_, in *W*, we use *F*_*kn*_ to denote the part of the objective function in (16) that is relevant only to *w*_*kn*_. It is easy to check that

(29) $$\begin{array}{l} F_{\text{kn}}^{\prime }={\left(\frac{\mathrm{\partial O}}{\mathrm{\partial W}}\right)}_{\text{kn}}=\left(-2H^{⊤}\text{diag}(-\rho^{t})X\right)\\ \;{\left(+2H^{⊤}\text{diag}(-\rho^{t})\text{HW}\right)}_{\text{kn}}\\ F_{\text{kn}}^{\prime \prime }={\left(\frac{\partial^{2}O}{\partial^{2}W}\right)}_{\text{kn}}=2{\left(H^{⊤}\text{diag}(-\rho^{t})H\right)}_{\text{kk}} \end{array}$$

Since the updating rule is essentially based on elements, it is sufficient to show that each *F*_*kn*_ is nonincreasing under the update step of (25).

#### Lemma 2

Function

(30) $$\begin{array}{l} \;G(w,w_{\text{kn}}^{t})=F_{\text{kn}}^{t}(w_{\text{kn}}^{t})+F_{\text{kn}}^{\prime }(w_{\text{kn}}^{t})(w-w_{\text{kn}}^{t})\\ \;+\frac{{\left(H^{⊤}\text{diag}(-\rho^{t})\text{HW}\right)}_{\text{kn}}}{w_{\text{kn}}^{t}}{\left(w-w_{\text{kn}}^{t}\right)}^{2} \end{array}$$

is an auxiliary function for *F*_*kn*_, which is relevant only to *w*_*kn*_.

#### Proof

Since *G*(*w*,*w*)=*F*_*kn*_(*w*) is obvious, we only need to show that $G(w,w_{\text{kn}}^{t})\geq F_{\text{kn}}(w)$. To do this, we compare the Taylor series expansion of *F*_*kn*_(*w*),

(31) $$\begin{array}{l} F_{\text{kn}}(w)=F_{\text{kn}}(w_{\text{kn}}^{t})+F_{\text{kn}}^{\prime }(w_{\text{kn}}^{t})(w-w_{\text{kn}}^{t})\\ \;+\frac{1}{2}F_{\text{kn}}^{\prime \prime }(w_{\text{kn}}^{t}){\left(w-w_{\text{kn}}^{t}\right)}^{2}\\ \;=F_{\text{kn}}(w_{\text{kn}}^{t})+F_{\text{kn}}^{\prime }(w_{\text{kn}}^{t})(w-w_{\text{kn}}^{t})\\ \;+{\left(H^{⊤}\text{diag}(-\rho^{t})H\right)}_{\text{kk}}{\left(w-w_{\text{kn}}^{t}\right)}^{2} \end{array}$$

with (30) to find that $G(w,w_{\text{kn}}^{t})\geq F_{\text{kn}}(w)$ is equivalent to

(32) $$\begin{array}{ll} \frac{{\left(H^{⊤}\text{diag}(-\rho^{t})\text{HW}\right)}_{\text{kn}}}{w_{\text{kn}}^{t}} & \geq {\left(H^{⊤}\text{diag}(-\rho^{t})H\right)}_{\text{kk}}\\ {\left(H^{⊤}\text{diag}(-\rho^{t})\text{HW}\right)}_{\text{kn}} & \geq {\left(H^{⊤}\text{diag}(-\rho^{t})H\right)}_{\text{kk}}w_{\text{kn}}^{t} \end{array}$$

We have

(33) $$\begin{array}{lll} \;{\left(H^{⊤}\text{diag}(-\rho^{t})\text{HW}\right)}_{\text{kn}} & =\overset{K}{\underset{l=1}{\sum }}{\left(H^{⊤}\text{diag}(-\rho^{t})H\right)}_{\text{kl}}w_{\text{ln}}w^{t}\; & \;\\ & \geq {\left(H^{⊤}\text{diag}(-\rho^{t})H\right)}_{\text{kk}}w_{\text{kn}}^{t}.\; \end{array}$$

Thus, (32) holds and $G(w,w_{\text{kn}}^{t})\geq F_{\text{kn}}(w)$. □

We can now demonstrate the convergence of **Theorem 1**.

#### Proof of Theorem 1

Replacing *G*(*w*,*w*^*t*^) in (27) by (30) results in the update rule

(34) $$\begin{array}{l} w_{\text{kn}}^{t+1}=w_{\text{kn}}^{t}-w_{\text{kn}}^{t}\frac{F_{\text{kn}}^{\prime }(w_{\text{kn}}^{t})}{2{\left(H^{⊤}\text{diag}(-\rho )HW^{t}\right)}_{\text{kn}}}\\ \;=w_{\text{kn}}^{t}\frac{{\left(H^{⊤}\text{diag}(-\rho )X\right)}_{\text{kn}}}{{\left(H^{⊤}\text{diag}(-\rho )HW^{t}\right)}_{\text{kn}}}. \end{array}$$

Since (30) is an auxiliary function, *F*_*kn*_ is nonincreasing under this update rule as in (25).

Similarly, we can also show that *O* is nonincreasing under the updating steps in (24).

## Experiments

### Datasets

To test the proposed algorithm, we carry out extensive experiments on six cancer-related gene expression datasets. The six datasets consist of five multi-class sets as used in [4,23] and one binary class set [24]. The descriptions of the six datasets are summarized in Table 1. In these datasets, besides the gene expression data samples, the labels are also given. They were obtained from the diagnosis results and reported in different studies [23].

**Table 1:** Summary of the six cancer gene expression datasets used to test the NMF-MCC algorithm

**Dataset name**	**Diagnostic task**	**Samples ( *N *)**	**Genes ( *D *)**	**Cancer Classes ( *K *)**	**Ref**
Leukemia	Acute myelogenous leukemia	72	5327	3	[25]
Brain Tumor	5 human brain tumor types	90	5920	5	[26]
Lung Cancer	4 lung cancer types and normal tissues	203	12600	5	[27]
9 Tumors	9 various human tumor types	60	5726	9	[28]
SRBCT	Small, round blue cell tumors	83	2308	4	[29]
DLBCL	Diffuse large B-cell lymphomas	77	5469	2	[24]

### Performance metric

The proposed NMF-MCC algorithm will be used to represent gene expression data for k-means clustering. The clustering results are evaluated by comparing the obtained label of each sample with the label provided by the dataset. The clustering accuracy is used to measure the clustering performance. Given a micro-array dataset containing *N* samples that belong to *K* classes, we assume that *K* is given in all the algorithms tested here. For each sample, *x*_*n*_, let *c*_*n*_ be the cluster label predicted by an algorithm and *r*_*n*_ be the cancer type label provided by the dataset. The accuracy of the algorithm is defined as:

(35) $$\text{Accuracy}=\frac{\sum_{n=1}^{N}I(r_{n},c_{n})}{N},$$

where *I*(*A*,*B*) returns 1 if *A*=*B* and 0 otherwise.

### Tested methods

We first compared the MCC with other loss functions between *X* and *HW* for the NMF algorithm on the cancer clustering problem, including *l*_2_ norm distance, KL distance [10], *α*-divergence [4], and earth mover’s distance (EMC) [12]. We further compared the proposed NMF-MCC algorithm with other NMF-based algorithms, including the penalized matrix decomposition (PMD) algorithm [7], the original NMF algorithm [22], the sparse non-negative matrix factorization (SNMF) algorithm [3], the non-smooth non-negative matrix factorization (nsNMF) algorithm [9] and the projected gradient kernel nonnegative matrix factorization (PGK-NMF).

### Results

**Figure 3:**
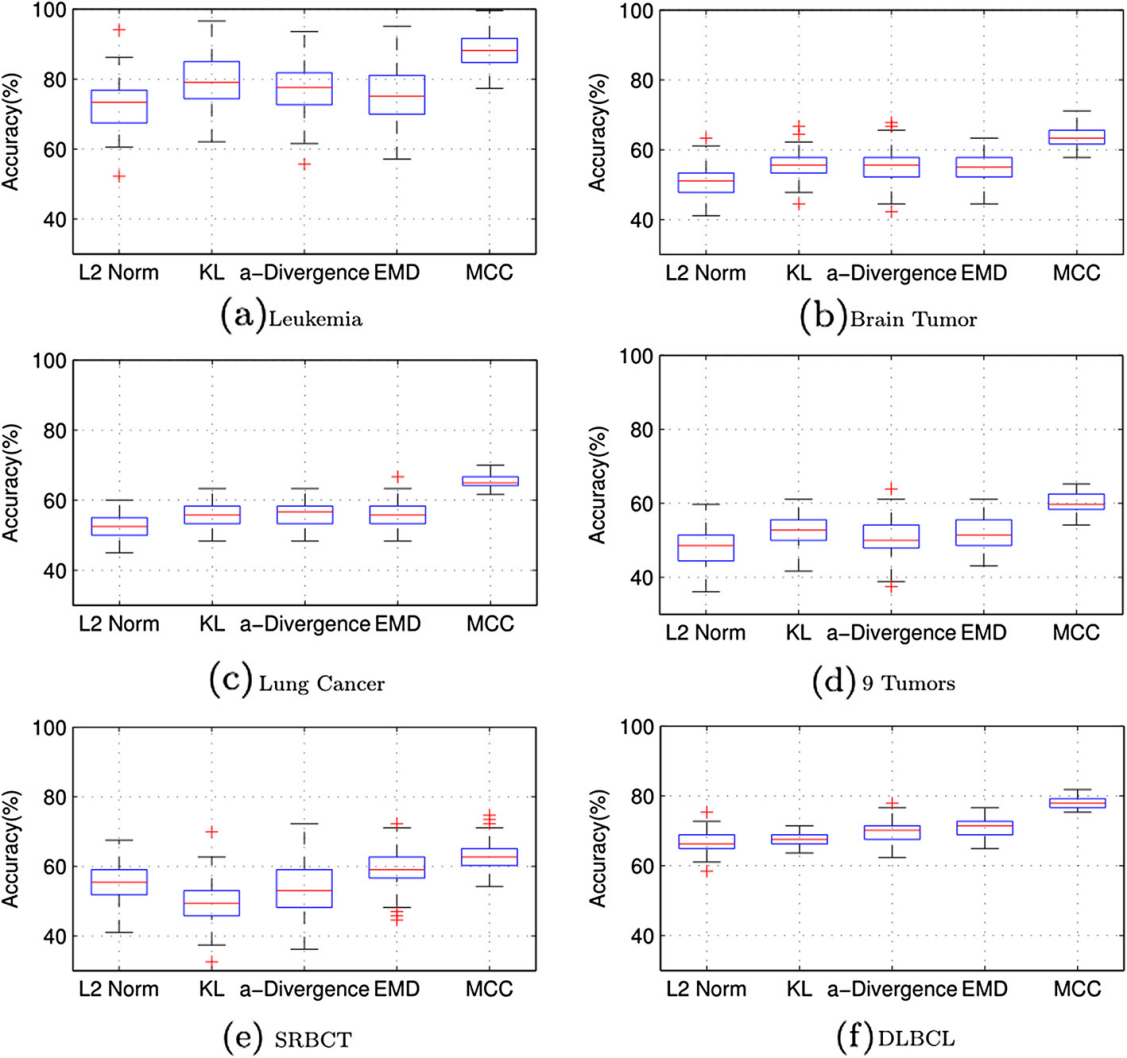
The boxplots of the clustering accuracies for NMF with different loss functions over 100 runs on the six gene expression datasets: (a) Leukemia, (b) Brain Tumor, (c) Lung Cancer, (d) 9 Tumors, (e) SRBCT, (f) DLBCL.

Since the initial *H* and *W* are selected randomly, we performed 100 independent trials and computed the average and the standard deviations of the accuracy for each loss function. The results from the comparison of MCC with other loss functions are presented in Figure 3. As shown in Figure 3, MCC consistently performed the best on all the six datasets. The other loss functions performed well on some datasets, but poorly on the others. It seems that the improvement of MCC increased when the number of genes increased. The standard deviation on the accuracy of MCC was much smaller than the standard deviation on the other loss functions, indicating that MCC is the most stable. On the other hand, EMD, although worked quite well in computer vision tasks [12], it did not perform well on gene expression data due to the significant difference between the image data and the gene expression data.

**Figure 4:**
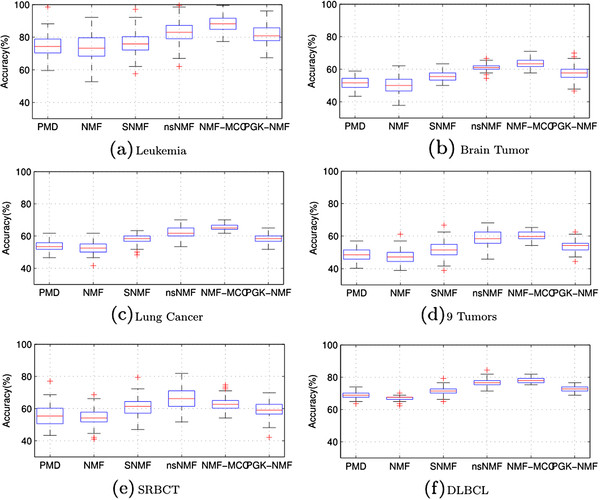
The boxplots of the clustering accuracies for different versions of NMF algorithms over 100 runs on the six gene expression datasets: (a) Leukemia, (b) Brain Tumor, (c) Lung Cancer, (d) 9 Tumors, (e) SRBCT, (f) DLBCL.

The results of the comparison of NMF-MCC with other related NMF methods are presented in Figure 4. Figure 4 shows the performance of different algorithms on the six datasets. The NMF-MCC algorithm outperformed the other algorithms on five out of the six datasets. The NMF-MCC algorithm could correctly cluster more than 88% and 78% of the samples in the Leukemia and DLBCL datasets, respectively, in a completely unsupervised manner. In contrast, the *l*_2_ norm distance-based NMF algorithm performed even worse than the baseline PMD algorithm on the Leukemia and DLBCL datasets, i.e., an average accuracy of 73% and 67%, respectively. This verifies that correntropy is a much better measure of cancer clustering data. Note that NMF-MCC significantly outperformed the other algorithms on the Lung Cancer dataset, which contains a large number of genes. This implies that among the large number of genes, only a small fraction is likely to be relevant to cancerous tumor growth or spread. In NMF-MCC, the auxiliary variables −***ρ*** acts as the feature selectors, we was able to select the relevant genes. Although the SNMF and nsNMF algorithms also improved on the performance of the baseline NMF algorithm, the improvement was much less than that of the NMF-MCC algorithm. A possible reason is that many genes exhibit similar patterns across all of the samples with only a few genes differentiating different cancer classes. They are likely to be sampled from a nonlinear manifold. Hence, the loss function defined by a linear kernel with either the *l*_2_ norm or the KL distance could not capture them. In contrast, the NMF-MCC algorithm had a loss function that was defined by the correntropy and a Gaussian kernel, which could capture the nonlinear manifold structure much more effectively. By mapping the gene expression data into the nonlinear dataspace by a Gaussian kernel, the PGK-NMF outperformed the original NMF. However, our NMF-MCC could even further improve the PGK-NMF by applying different kernels to different features.

**Figure 5:**
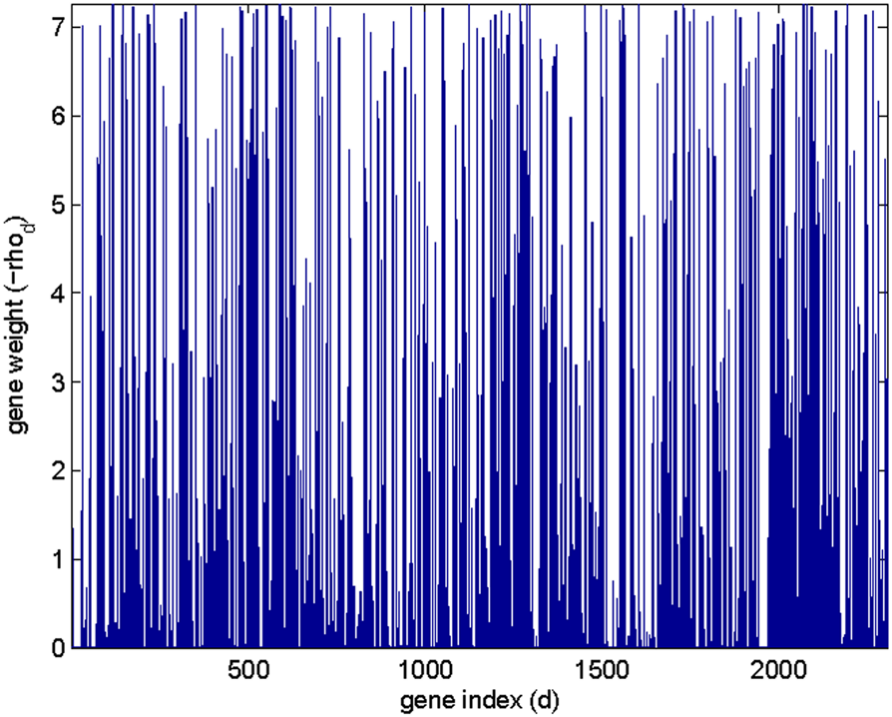
**The gene weight vector learned by NMF-MCC with *−ρ *on the SRBCT dataset.**

**Figure 6:**
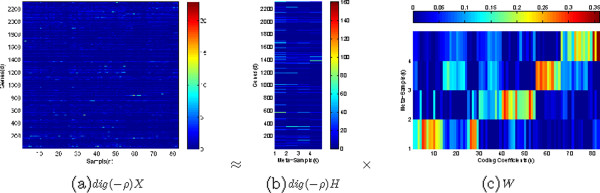
**The meta-sample matrix, *H, *weighted by *dig(−ρ*)** and the corresponding coding matrix,***W*, obtained from the NMF-MCC algorithm for the SRBCT dataset.**

To understand what genes were selected by the NMF-MCC algorithm, we drew the gene weight figure on the SRBCT dataset (Figure 5). It can be seen that the −***ρ*** vector is sparse, which shows the significance of certain genes. The resulting meta-sample matrix weighted by −***ρ*** with the corresponding coding matrix is shown in Figure 6. By comparing to the coding matrix learned by the original NMF with the *l*_2_ norm distance in Figure 1, we determine that the coding matrix learned by the NMF-MCC algorithm is much more discriminative among different cancer classes. On this dataset, the NMR-MCC algorithm achieved an average clustering accuracy of 63%.

## Discussion

Traditional unsupervised learning techniques select features with features selection algorithms and then do clustering using the selected features. The NMF-MCC algorithm proposed here achieves both goals simultaneously. The learned gene weight vector reflects the importance of the genes in the gene clustering task, and the coding matrix encodes the clustering results for the samples.

Our experimental results demonstrate that the improvement of NMR-MCC over the other methods increases when the number of genes increases. This shows the ability of the proposed algorithm to effectively select the important genes and cluster samples. This is an important property because high-dimensional data analysis has become increasingly frequent and important in diverse fields of sciences and engineering, and social sciences, ranging from genomics and health sciences to economics, finance and machine learning. For instance, in genome-wide association studies, hundreds of thousands of SNPs are potential covariates for phenotypes such as cholesterol level or height. The large number of features presents an intrinsic challenge to many classical problems, where usual low-dimensional methods no longer apply. The NMF-MCC algorithm has been demonstrated to work well on the datasets with small numbers of samples but large numbers of features. It can therefor provide a powerful tool to study high-dimensional problems, such as genome-wide association studies.

## Conclusion

We have proposed a novel NMF-MCC algorithm for gene expression data-based cancer clustering. Experiments demonstrate that correntropy is a better measure than the traditional *l*_2_ norm and KL distances for this task, and the proposed algorithm significantly outperforms the existing methods.

## Competing interests

The authors declare that they have no competing interests.

## Authors’ contributions

JW designed and implemented the algorithm, conducted the experiments, performed data analysis and drafted the manuscript. XW revised the manuscript. XG supervised the study and drafted the manuscript. All authors read and approved the final manuscript.
